# Vaccine adjuvants: mechanisms and platforms

**DOI:** 10.1038/s41392-023-01557-7

**Published:** 2023-07-19

**Authors:** Tingmei Zhao, Yulong Cai, Yujie Jiang, Xuemei He, Yuquan Wei, Yifan Yu, Xiaohe Tian

**Affiliations:** 1 Laboratory of Aging Research and Cancer Drug Target, State Key Laboratory of Biotherapy and Cancer Center, National Clinical Research Center for Geriatrics, West China Hospital, Sichuan University, No. 17, Block 3, Southern Renmin Road, Chengdu, 610041 Sichuan People’s Republic of China; 2 Division of Biliary Tract Surgery, Department of General Surgery, West China Hospital, Sichuan University, Chengdu, China; 3 Department of Radiology and Huaxi MR Research Center (HMRRC), Functional and Molecular Imaging Key Laboratory of Sichuan Province, West China Hospital, Sichuan University, Chengdu, China

**Keywords:** Vaccines, Antigen processing and presentation, Drug delivery, Nanobiotechnology, Nanobiotechnology

## Abstract

Adjuvants are indispensable components of vaccines. Despite being widely used in vaccines, their action mechanisms are not yet clear. With a greater understanding of the mechanisms by which the innate immune response controls the antigen-specific response, the adjuvants’ action mechanisms are beginning to be elucidated. Adjuvants can be categorized as immunostimulants and delivery systems. Immunostimulants are danger signal molecules that lead to the maturation and activation of antigen-presenting cells (APCs) by targeting Toll-like receptors (TLRs) and other pattern recognition receptors (PRRs) to promote the production of antigen signals and co-stimulatory signals, which in turn enhance the adaptive immune responses. On the other hand, delivery systems are carrier materials that facilitate antigen presentation by prolonging the bioavailability of the loaded antigens, as well as targeting antigens to lymph nodes or APCs. The adjuvants’ action mechanisms are systematically summarized at the beginning of this review. This is followed by an introduction of the mechanisms, properties, and progress of classical vaccine adjuvants. Furthermore, since some of the adjuvants under investigation exhibit greater immune activation potency than classical adjuvants, which could compensate for the deficiencies of classical adjuvants, a summary of the adjuvant platforms under investigation is subsequently presented. Notably, we highlight the different action mechanisms and immunological properties of these adjuvant platforms, which will provide a wide range of options for the rational design of different vaccines. On this basis, this review points out the development prospects of vaccine adjuvants and the problems that should be paid attention to in the future.

## Introduction

**Fig. 1:**
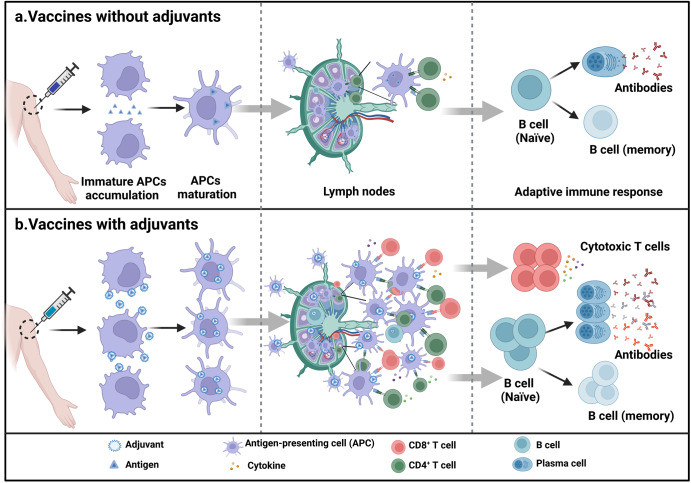
Adjuvants enhance the immunogenicity of vaccines. **a** Vaccines without adjuvants induce modest production of T helper-polarizing cytokines, antibodies, and activated T cells. **b** In contrast, vaccines with adjuvants promote the maturation of more APCs, increase the interaction between APCs and T cells, promote the production of greater numbers and more types of T helper-polarizing cytokines, multifunctional T cells, and antibodies, leading to broad and durable immunity, as well as dose and antigen savings. This figure was created with BioRender (https://biorender.com/)

**Fig. 2:**
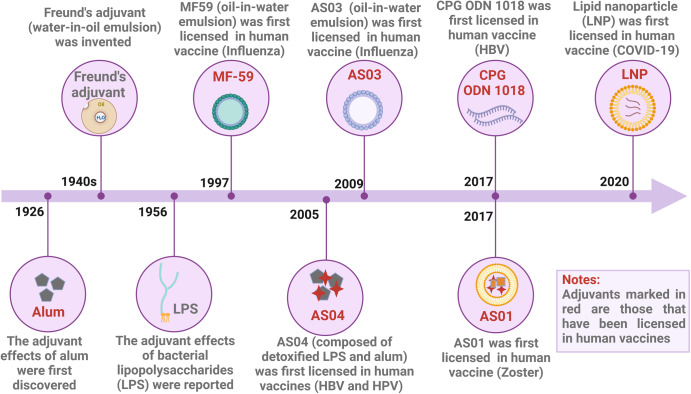
Timeline of major events in the research history of vaccine adjuvants. This figure was created with BioRender (https://biorender.com/)

Adjuvants are defined as various components that enhance the immunogenicity of vaccines when administered in conjunction with vaccine antigens (Fig. 1).^1^ Adjuvants can range from synthetic small molecule compounds to complex natural extracts and particulate materials.^2^ The first evidence of adjuvants appeared in 1926, when Alexander Glenny found that mixing aluminum salts with antigens and injecting them into guinea pigs induced more antibodies than administering antigens alone (Fig. 2).^3^ Subsequently, in the 1940s, Freund and his colleagues developed water-in-oil emulsions, which led to the creation of Freund’s adjuvants.^4,5^ However, Freund’s adjuvant is not licensed for use in human vaccines because of its toxicity to humans. Similar to Freund’s adjuvants, the use of bacterial lipopolysaccharide (LPS) adjuvants in human vaccines has been limited due to their local and systemic side effects. In fact, from the 1920s through the 1990s, only aluminum adjuvants were licensed, despite efforts to develop new adjuvants for human vaccines. It was not until 1997 that the oil-in-water emulsion MF59 was licensed in Europe as an adjuvant for influenza vaccines. In the following 20 years, four other adjuvants (AS04, AS03, AS01, and CpG ODN 1018) were licensed for use in vaccines, which changed the monotony of adjuvants for human vaccines.^6^ In addition, many other different classes of compounds had been evaluated as adjuvants during this time, including mineral salts, microbial products, emulsions, saponins, synthetic small molecule agonists, polymers, nanoparticles, and liposomes.^7^ They have shown to enhance the strength, breadth, and persistence of immune responses in preclinical and clinical studies.^8^

Although adjuvants have long been widely used in vaccines, the mechanisms by which they enhance immune responses have not been well characterized. It was not until the revelation of the mechanism by which the innate immune response controls the adaptive immune response that scientists began to understand the action mechanisms of adjuvants.^9^ Adjuvants guide and enhance specific adaptive immune responses by targeting innate immune cells and activating pattern recognition receptors (PRRs) signaling pathways.^10^ Subsequently, it was discovered that some delivery materials can also act as adjuvants by enhancing adaptive immune responses through mimicking the size or spatial structure of natural pathogens to facilitate antigen uptake and presentation by antigen-presenting cells (APCs).^11^

Despite the above advances in the development of adjuvants, there is little systematic generalization and summary of the action mechanisms of adjuvants due to their broad definitions and complex mechanisms. Moreover, due to the lack of systematic and in-depth understanding of the mechanisms, characteristics, immune effectiveness, and application scenarios of the current adjuvant platforms, it is difficult to match and design appropriate adjuvants for specific vaccines. This has led current vaccines gradually showing shortcomings in their use, such as the inability to provide long-term protective immunity, weak immunity in elderly populations, and the inability to provide effective cellular immunity.^12,13^ Therefore, in order to solve these problems, this review attempts to summarize the mechanisms of adjuvants firstly. Then, the mechanisms, characteristics and application progress of the classical adjuvant platforms and the adjuvant platforms under investigation with potential development value, are summarized and described. In the final part of this paper, some possible future directions of adjuvants are discussed. This review is expected to provide an effective reference for further research on the mechanisms of adjuvants, rational use of existing adjuvants, and design and development of new adjuvants.

## The mechanisms of adjuvants

**Fig. 3:**
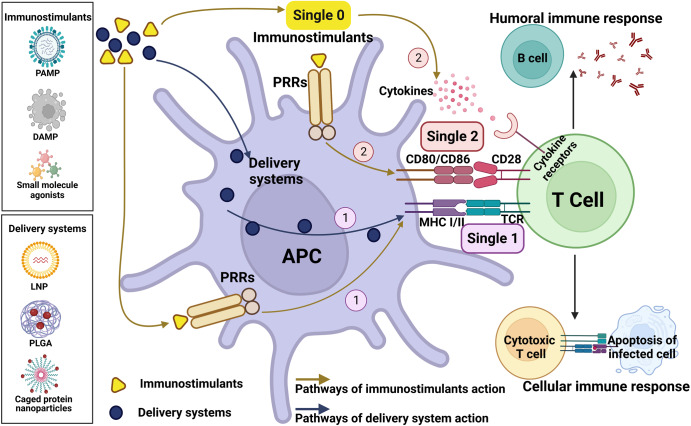
The core of the action mechanisms of adjuvants. Adjuvants are classified as immunostimulants and delivery systems. Immunostimulants such as PAMPs, DAMPs, and chemically synthesized small molecule agonists, provide danger signals (signal 0) to activate PRRs on APCs, thereby enhancing antigen presentation on MHC molecules (signal 1). In addition, activation of PRRs leads to upregulation of cytokines and co-stimulatory molecules expression, which results in enhanced co-stimulatory signaling (signal 2). Delivery systems such as LNPs, PLGA, and self-loaded protein nanoparticles, act by facilitating the presentation of antigens on MHC molecules (signal 1). This figure was created with BioRender (https://biorender.com/)

Adjuvants have been widely used in vaccines to promote the success of vaccination. Adjuvants enhance the adaptive immunity of vaccines by activating innate immune cells.^10^ The core concept is that adjuvants promote the generation of antigen presentation signals (signal 1) and co-stimulatory signals (signal 2) by activating APCs (Fig. 3). The antigen presentation signals are the antigen peptides-major histocompatibility complexes (MHC) that are presented on the surface of APCs after antigens have been taken up and processed. Co-stimulatory signals include co-stimulatory molecules (e.g., CD40, CD80, CD86) expressed on the surface of APCs and secreted inflammatory cytokines (e.g., IL-6, IL-10, IL-12, and TNF-α). The production of these two signals can strongly induce the activation of naive T cells, leading to an enhanced adaptive immune response.^14,15^ Immunostimulants, such as pathogen-associated molecular patterns (PAMPs), damage-associated molecular patterns (DAMPs), and chemically synthesized small molecule agonists of TLRs, can lead to signal 1 and signal 2 production by APCs. Delivery systems, such as lipid nanoparticles (LNPs), poly(lactide-*co*-glycolide) (PLGA), and caged protein nanoparticles, play a role by facilitating the presentation of antigens on MHC molecules (signal 1). Notably, some studies have shown that many nanoparticle delivery systems can directly target B cells to induce an optimally effective antibody response.^1,7,11^ In the following, we describe in detail the specific mechanisms by which immunostimulants and delivery systems exert their adjuvant efficacy.

### The mechanisms of immunostimulants

**Fig. 4:**
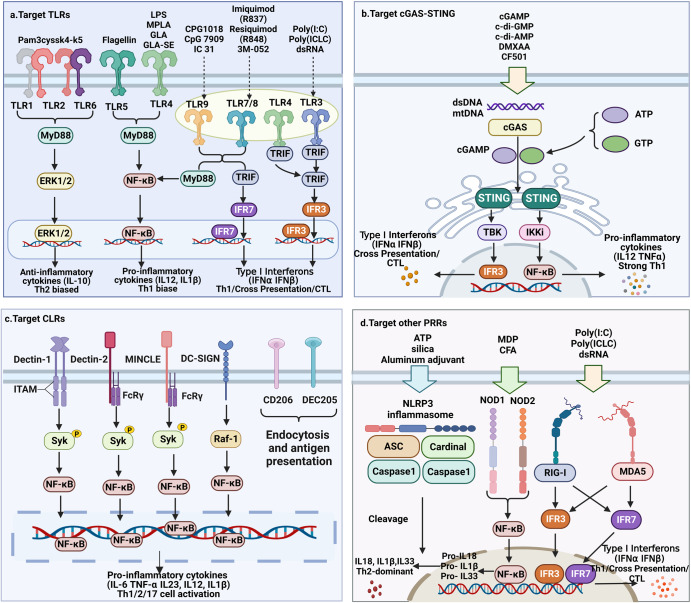
The simplified diagram of immunostimulants regulating adaptive immune responses by activating PRRs. Different types of immunostimulants send signals through different PRRs, leading to different cytokines secretion, thereby inducing different adaptive immune responses. Immunostimulants induce and modulate adaptive immune responses by targeting and activating **a** TLRs; **b** cGAS-STING; **c** CLRs; **d** Other PRRs. This figure was created with BioRender (https://biorender.com/)

Based on the mechanisms of action, adjuvants can be classified as immunostimulants and delivery systems. Immunostimulants are danger signal molecules that lead to the maturation and activation of APCs by targeting specific receptors on APCs cells. Specifically, immunostimulants act as PAMPs, DAMPs or their mimics, which can interact with PRRs on APCs to trigger an innate immune response and lead to the activation and maturation of APCs. Mature APCs terminate their phagocytic antigen activity and enhance their ability to present antigens and express high levels of co-stimulatory signals and cytokines.^16^ This leads to the initiation and enhancement of the adaptive immune responses. Furthermore, it is noteworthy that different types of immunostimulants will signal through different PRRs and lead to different cytokines secretion, which is a major determinant of the adaptive immune responses (Fig. 4).^17,18^

#### Targeting TLRs pathway

Initially, it is found that immunostimulants exert their effects by interacting with TLRs on APCs (Fig. 4).^19^ Agonists of TLRs as adjuvants have shown promising results by activating TLRs that lead to enhanced antigen presentation, upregulation of co-stimulatory signals and cytokine expression, and ultimately to enhanced adaptive immune responses.^20–23^ Notably, different types of immunostimulants mediate different adaptive immune responses by signaling with different TLRs.^24^ Overall, since TLR1, TLR2, TLR4, TLR5, and TLR6 are expressed on the cell surface, they mainly recognize microbial membrane components such as lipids, lipoproteins, and proteins. Thus, activation of TLRs on the surface of APCs by the corresponding immunostimulants usually leads to the production of pro-inflammatory cytokines such as IL-1β, TNF-α, and IL-6, and ultimately leads to the production of Th1 or Th2 type immune responses.^19,25^ TLR3, TLR7, TLR8, and TLR9 are intracellular TLRs that are expressed in intracellular vesicles and react with nucleic acids.^26^ Activation of these intracellular TLRs by the corresponding types of immunostimulants usually leads to the production of type I interferons.^27^ Type I interferons can promote the differentiation of Th1 cells and regulate their function. In addition, type I interferons promote antigen cross-presentation to CD8^+^ T cells via conventional dendritic cells (DCs) and may directly stimulate the proliferation of CD8^+^ T cells.^28^ The specific mechanisms by which immunostimulants act through TLRs are as follows, (1) immunostimulants bound to TLR2 heterodimers (TLR2/1 or TLR2/6) initiate signaling through the myeloid differentiation primary response 88 (MyD88) pathway and activate NF-κB, thereby inducing the production of pro-inflammatory cytokines such as IL12. IL-12 is involved in the differentiation of naive T cells to Th1 cells.^29,30^ In addition, immunostimulants targeting TLR2 also lead to enhanced extracellular signal-regulated kinase 1/2 (ERK1/2) signaling, resulting in enhanced expression of c-Fos protein, which will suppress IL-12 expression and enhance IL-10 expression. This drives naive T cells to polarize into Th2-type cells.^25^ Thus, immunostimulants targeting TLR2 mainly induce Th2-type adaptive immune responses. (2) Immunostimulants bound to TLR3 initiate signaling through the toll/interleukin-1 receptor domain-containing adapter inducing interferon-β (TRIF) pathway, activating interferon regulatory factor 3 (IRF3) and stimulating APCs to produce type I interferons. This ultimately leads to Th1-type responses and cytotoxic T lymphocytes (CTLs) production. (3) Immunostimulants bound to TLR4 and TLR5 initiate signaling via the MyD88 pathway to activate NF-κB, which induces pro-inflammatory cytokine secretion and drives naive T cells to Th1-type cell polarization. In addition, it is noteworthy that immunostimulants triggering adaptive immunity via TLR4 can also signal through TRIF, leading to the activation of IRF3, resulting in the production of small amounts of type I interferons.^31^ (4) Immunostimulants targeting TLR7/8/9 located in the endosome activate NF-κB and interferon regulatory factor 7 (IRF7) via the MyD88 pathway, trigger the production of pro-inflammatory cytokines and type I interferons by APCs, and induce strong Th1 and CTLs responses.

#### Targeting cGAS-STING pathway

With the revelation of the unique role of the cyclic guanosine monophosphate-adenosine monophosphate synthase-stimulator of interferon genes (cGAS-STING) pathway in coordinating innate and adaptive immunity, there has been a growing interest in scientists trying to target agonists of the cGAS-STING pathway as vaccine adjuvants.^32^ cGAS is a cytoplasmic DNA receptor that can be activated by double-stranded DNA. cGAS, when activated, loops cytoplasmic adenosine monophosphate and guanosine monophosphate into cyclic guanosine monophosphate-adenosine monophosphate (cGAMP).^33^ Subsequently, cGAMP locks into the v-shaped binding pocket of the STING dimer, causing conformational changes, aggregation, and activation of STING. After that, NF-κB and IRF3 will be activated, which in turn promotes the production of pro-inflammatory cytokines and type I interferons.^34^ Type I interferons such as IFN-α/β can induce the maturation of APCs, upregulate co-stimulatory signals and enhance their ability to present or cross-present antigens.^35^ Thus, immunostimulants targeting the cGAS-STING pathway not only polarize naive T cells into Th1-type cells, but also promote the production of CTLs (Fig. 4). Immunostimulants targeting cGAS-STING include nucleotide small molecule agonists and non-nucleotide small molecule agonists. Among them, nucleotide small molecule agonists are usually natural ligand molecules based on cyclic dinucleotides (CDNs), such as cyclic dimeric guanosine monophosphate (c-di-GMP), cyclic dimeric adenosine monophosphate (c-di-AMP), 2’,3’-cGAMP and 3’,3’-cGAMP.^32^ And examples of non-nucleotide small molecule agonists are DMXAA and CF501.^36,37^ In addition, it is noteworthy that mitochondrial DNA (mtDNA) in the cytoplasm can also be sensed by cGAS, thus activating the STING pathway.^38^

#### Targeting CLRs pathway

C-type lectin receptors (CLRs) are a superfamily including Dectin-1, Dectin-2, MINCLE, DC-SIGN, CD206, CD205, and so forth. CLRs are mostly localized on cell membranes and function as antigen receptors involved in capturing and presenting antigens.^39,40^ Immunostimulants with a carbohydrate structure generally activate CLRs, which subsequently induce APCs to initiate the internalization, processing and presentation of antigens to enhance the development of adaptive immune responses.^41,42^ In addition, some immunostimulants can activate CLRs, which trigger different signaling pathways, induce the expression of specific cytokines, and thus control the polarization direction of naive T cells.^43^ For example, the activation of Dectin-1 by β-glucan leads to the phosphorylation of Syk kinase, which in turn activates NF-κB, leading to the manufacture of the pro-inflammatory cytokines IL12, IL-1β, IL6, and IL23, thereby inducing the differentiation of naive T cells into Th1 and Th17 cells (Fig. 4).^44^ Type 1 Th cells induce a strong CTLs response as well as drive the conversion of immunoglobulins in B cells to IgG2a. Th17 effector cells are characterized by the production of IL-17, IL-17F, and IL-22 and trigger a massive inflammatory response by recruiting neutrophils.^45^ Activation of Dectin-2 and MINCLE and DC-SIGN by immunostimulants can also lead to NF-κB activation and pro-inflammatory cytokine production. However, since Dectin-2 and MINCLE do not possess immunoreceptor tyrosine-based activation motifs (ITAMs), it is necessary for them to bind to FcRγ, which carries ITAM, for signal transduction to occur.^46^ Notably, stimulation of DEC-205 and CD206 by polysaccharide ligands is usually biased toward inducing enhanced phagocytosis and antigen presentation, but not intracellular signal transduction.

#### Targeting other PRRs

In later studies, scientists found that immunostimulants could also activate a number of other PRRs, such as nucleotide-binding oligomerization domain 1 (NOD1), nucleotide-binding oligomerization domain 2 (NOD2), NOD-like receptor thermal protein domain associated protein 3 (NLRP3), retinoic acid-induced gene I (RIG-I), and melanoma differentiation-associated gene 5 (MDA5) (Fig. 4). NOD1, NOD2, and NLRP3 are members of the nucleotide-binding oligomerization domain-like receptors (NLRs) family.^47^ Activation of them by adjuvants will lead to upregulation of MHC II by APCs and contribute to enhanced antigen presentation.^23,48^ In addition, activation of these NLRs will lead to the production of pro-inflammatory cytokines such as IL-1β and IL-18, driving naive T cells to bias polarization into Th2 type cells.^48^ Muramyl dipeptide (MDP) or complete Freund’s adjuvant (CFA) can activate NOD1 and NOD2 and subsequently stimulate NF-κB transcriptional activation to produce IL1, IL18, and IL33 precursors.^23^ Subsequently, in the presence of the activated NLRP3 inflammasome complex, caspase1 cleaves these factors to their active forms.^49^ The NLRP3 inflammasome complex consists of NLRP3 protein, apoptosis-associated speck-like protein (ASC), cardinal and caspase1 proteins.^50^ Overall, activation of NOD1, NOD2, and NLRP3 will result in the generation of a predominantly Th2 type of immune response. RIG-I and MDA5 are members of the retinoic acid-inducible gene I-like receptors (RLRs) family, which mainly recognize RNA.^51^ Most TLR3 agonists, such as poly-I:C, also activate MDA5 in the APCs. Activation of RIG-I and MDA5 leads to activation of IRF3 and IRF7, which in turn induces type I interferons expression.^52^ Thus, immunostimulants targeting these two receptors are ultimately biased towards leading to the production of Th1 cells and CTLs.^53^

### The mechanisms of delivery systems

Delivery systems are defined as carrier materials that load antigens and increase the uptake and presentation of antigens by APCs. In other words, the primary function of the delivery system is to facilitate antigen presentation. The antigen presentation process involves the recognition, uptake, and internalization of antigens by APCs, followed by loading and presentation on the surface of APCs by the MHC.^54^ The delivery systems can increase the antigen signals to be presented on the surface of APCs in one or more ways.

#### Prolonging the bioavailability of antigens

**Fig. 5:**
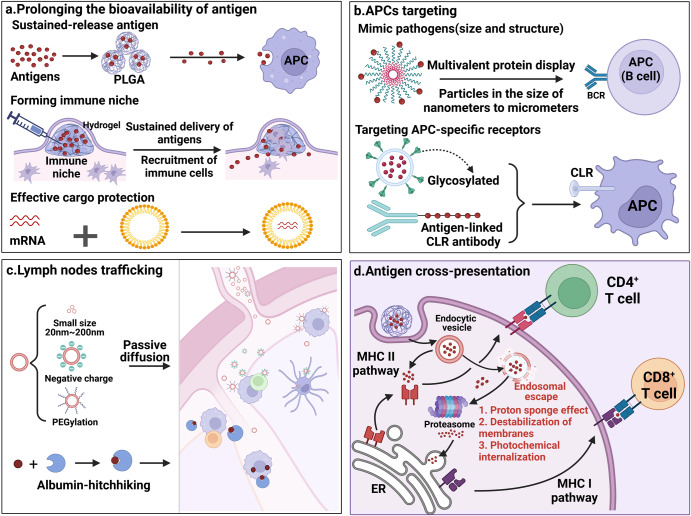
The diagram of the action mechanisms of the delivery systems. Delivery systems promote enhanced antigen presentation or antigen cross-presentation to enhance adaptive immune responses by **a** prolonging the bioavailability of antigens; **b** targeting antigens to APCs; **c** trafficking antigens directly to lymph nodes; and **d** promoting endosomal escape. This figure was created with BioRender (https://biorender.com/)

The delivery systems achieve prolonging antigens bioavailability by (1) allowing a sustained release of antigens, (2) forming immune niches, and (3) providing cargo protection for antigens (Fig. 5). The prolonged antigens bioavailability will ensure that there are sufficient antigens available to the APCs. Accordingly, this will lead to more MHC-antigen peptides signal production.

Delivery systems can prolong the bioavailability of antigens in the immune system by sustainably releasing antigens.^55^ For example, one of the action mechanisms of nanoemulsion delivery systems (e.g., MF59, AS03) has been thought to be sustained antigen release.^56^ Recently, some delivery systems under investigation have also been found to have the ability to slowly release antigens such as injectable hydrogels.^57^ The injectable hydrogel allows the APCs to continuously present the antigen through slow release, which facilitates the germinal center B cells to undergo multiple rounds of affinity selection, ultimately leading to the production of larger quantities and high-affinity antibodies.^58–60^ Notably, the sustained release time of the vaccine components in the hydrogel can be controlled by adjusting the size of the hydrogel grid, thus tuning the release time window.^61^ In addition, solid polymer particles made of biodegradable PLGA or polyanhydride polymers can be used to encapsulate antigens and slowly release subunit antigens over a period of days to months.^62,63^ We also noticed that self-assembled scaffolds with macroporous structures also have the ability to slowly release antigens.^64^ Not only that, self-assembled scaffolds can release inflammatory signals and enhance serum antibody levels and CTLs levels by loading immunostimulants in addition to antigens.^65^ In summary, the use of sustained-release techniques to extend the exposure time and bioavailability of antigens and adjuvants in the immune systems can improve the potency, durability, and quality of adaptive immune responses.

In addition, some delivery systems can achieve higher immune activation by forming an antigen depot/immune niche at the injection site. Delivery systems that function in this way not only prolong the retention time of antigens in the immune system, but also recruit more immune cells to infiltrate the injection site, which will increase the uptake of antigens and provide local inflammatory cues to activate the subsequent adaptive immune responses.^66^ Delivery systems that generate an inflammatory immune niche have been shown to significantly prolong the bioavailability of antigens, increase the uptake of antigens, recruit immune cells, and enhance the antibody titers and cellular immune responses.^65,67–70^ The infiltration of innate immune cells into the injection site allows niche-forming delivery materials to have a greater immune activation capacity than delivery materials prepared by sustained-release techniques alone.^71^ In order to infiltrate immune cells into the delivery system, some cytokines can be incorporated into the delivery system and released slowly, thereby increasing cell migration. The most commonly used cytokine is granulocyte-macrophage colony-stimulating factor (GM-CSF). Recently, Sun et al. developed an injectable poly(caprolactone)-poly(ethylene glycol)-poly(caprolactone) thermosensitive hydrogel encapsulating GM-CSF and ovalbumin, which increased DCs recruitment at the injection site and improved the efficiency of antigens uptake.^67^ Besides, encapsulating some immunostimulants such as TLR agonists can also promote the recruitment and infiltration of innate immune cells.^72^ In addition, studies have found that some self-adjuvant delivery materials can also promote immune cell infiltration. For example, the mesoporous silica rod is a self-adjuvant delivery material that can activate the NLRP3 innate immune pathway. When injected into mice, it increases innate immune cell infiltration to the injection site and enhances immune responses.^73^

Moreover, delivery systems can also prolong the bioavailability of antigens by protecting them (especially DNA and mRNA) from breakdown by enzymes in the body thus maintaining their bioactivity. For example, mRNA antigens are susceptible to degradation by RNA enzymes in extracellular serum. Therefore, they need to be encapsulated in a sealed carrier to avoid enzymatic hydrolysis and ensure the smooth delivery of mRNA to the target cells. LNPs are a kind of proven effective mRNA delivery system, and one of their basic functions is to encapsulate mRNA and protect it from degradation RNA enzymes.^74–76^

Notably, since an adjuvant has multiple mechanisms, prolonging the bioavailability of antigens is only one of the mechanisms, and how much it accounts for all the action mechanisms of an adjuvant needs to be carefully studied. For example, the sustained-release of antigens through the formation of an “antigen depot” to prolong the retention and bioavailability of antigens is traditionally considered a key mechanism of action for aluminum adjuvants.^77^ However, with the deepening of studies, some researchers found that removing the “antigen depot” after aluminum adjuvant administration did not significantly reduce the production of antigen-specific T and B cell responses.^78^ This implies that the mechanism of forming an antigen depot to retain antigens may not be the key action mechanism of aluminum adjuvants and that the action mechanisms of adjuvants should be fully and accurately understood for more rational use of adjuvants and development novel adjuvants.

#### APCs targeting

Delivery systems can target APCs by following two approaches and enable the effective uptake of antigens by APCs (Fig. 5). Increased antigen uptake, in turn, increases the amounts of MHC-antigen peptides presented on the APCs surface, leading to a stronger adaptive immune response.

The first approach is that delivery systems can facilitate antigens uptake by APCs by mimicking the size dimensions and spatial structure of pathogens. Since viruses and bacteria are nano to micron-sized particles, the immune system has evolved to recognize and respond to particulate antigens. Microscale or nanoscale materials have been used to deliver antigens, increasing the sizes of the antigens and thus improving the uptake by APCs to some extent.^79^ For example, encapsulation of antigens with a liposomal nanoparticle improves recognition and endocytosis of antigens by APCs compared to soluble antigens, leading to increased antigen presentation and induction of higher magnitude and higher affinity antibodies.^80^ Another example is that a novel hydrogel microparticle delivery system loaded with the H5N1 influenza antigen can improve humoral responses and enhance T-cell activation.^81^ One of the reasons for this is that hydrogel microparticles increase the size of the antigen which in turn improves the recognition and uptake of the antigen by APCs. Moreover, highly ordered and repetitive spatial structures are inherent to pathogens, and the immune systems have evolved to recognize and respond to such structures with great sensitivity.^82^ Self-assembled protein nanoparticles and other polyvalent particle delivery systems display multivalent antigens to APCs by mimicking the highly ordered and repetitive spatial structure of pathogens, increasing the probability of antigen uptake by APCs.^83–86^ It was found that these delivery systems elicit strong humoral and cellular immune responses even in the absence of supplementation with other immunostimulants.^87–89^ Notably, the highly ordered and repetitive spatial structure of the delivery systems is highly conducive to the co-aggregation of B cell receptors (BCRs), which can lead to strong activation of B cells and ultimately to the production of high-affinity antibodies and memory B cells.^87,90^

In addition, delivery systems promote more efficient antigen uptake by directly targeting specific receptors (e.g., Fc receptors, CLRs) on APCs. Numerous studies have shown that delivery systems targeting immunogens to Fc receptors on APCs can selectively enhance antigen uptake and cellular immunity in vitro and in vivo.^91–94^ In addition, CLRs are a class of PRRs that are specifically expressed on a subgroup of DCs. Members of this family include DEC-205, DC-SIGN, and mannose receptors, among others. The use of CLRs ligands (usually carbohydrates) to modify the delivery system will result in more direct targeting of antigens to specific DCs, thus enhancing antigen uptake.^95^ For example, mannose-modified polymers for delivery of melanoma antigenic peptides strongly induce prophylactic and therapeutic antitumor immune responses in melanoma models.^96^ Another example, CDX-1401 is a vaccine consisting of a DEC-205-specific monoclonal antibody fused to the full-length tumor antigen NY-ESO-1. Studies have shown that this vaccine enhances antitumor effects by specifically targeting DCs via the DEC-205 receptor, leading to increased antigen uptake and presentation.^97^ It has also shown promising clinical value when combined with other drugs (NCT02129075).

Considering that different subpopulations of DCs associated with lymph nodes may have different functions in inducing different types of T cell responses,^98^ the targeted immune cell populations of delivery systems (especially nanoparticles) are a key consideration in the development of vaccine delivery systems. From the above description of APCs targeting, it can be seen that the surface of the nanoparticle delivery systems can be specifically modified to target specific cell populations actively. Firstly, nanoparticles can be modified with functionalized ligands to target specific cell populations. For example, by coupling an antibody against CD169 to the surface of nanoparticles, the nanoparticles can be targeted to subcapsular sinus macrophages.^99^ By coupling the CLEC9A antibody with nanoparticles, the nanoparticles can preferentially target conventional type 1 DCs (cDC1).^100,101^ Secondly, adjusting the sizes of nanoparticles is also a strategy to target specific cell populations. When nanoparticles are designed to remain at 50–100 nm, they will preferentially target follicular dendritic cells (FDCs) and be retained in the FDCs.^102,103^ This is important for the generation of high-affinity and durable antibody immune responses.^104^ Furthermore, increasing the antigenic valence of nanoparticles and glycosylation modification of nanoparticles will also allow the nanoparticles to preferentially target FDCs.^84^ In addition, nanoparticles can be designed for displaying multivalent antigen proteins to target and activate B cells.^90^ In summary, these approaches allow nanoparticles to actively target different cell populations.

#### Lymph node trafficking

A large number of innate immune cells and lymphocytes accumulate in the lymph nodes, which are the site of the initial immune responses.^105^ Thus, lymph nodes are key targets for delivery systems. Some delivery systems can facilitate antigen trafficking to the lymph nodes directly, thereby increasing the chances of antigens encountering APCs in the lymph nodes, resulting in more antigens uptake and presentation by APCs and an increase in antigen presentation signals. Delivery systems traffic antigens to lymph nodes directly in two ways (Fig. 5).

In the first approach, the delivery systems are transported directly to the lymph nodes by passive diffusion. Delivery systems with suitable dimensions and surface properties (such as charge and hydrophobicity) can enter the afferent lymphatics by passive diffusion and subsequently enter the lymph nodes.^11^ Studies have shown that the optimal size is between 5 to 100 nm.^106^ If the size of the delivery system is too small, it tends to enter the capillaries. Whereas if the size of the delivery vehicle adjuvant is too large, it may not pass through the gap between the endothelial cells of the afferent lymphatic vessels and thus cannot enter the afferent lymphatic vessels. Besides, in this approach, the delivery system is optimally charged to carry a net negative charge, rather than a net positive charge, since the interstitial matrix of the afferent lymphatic vessels is composed mainly of collagen fibers and negatively charged glycosaminoglycans.^107^ In addition, the hydrophilicity of the delivery systems affects their efficiency in transporting vaccines between afferent lymphatic vessels.^108^ For example, the increased hydrophilicity of PEG-modified 50 nm polymers resulted in more particle accumulation in the lymph nodes of rats after subcutaneous injection compared to non-PEG-modified particles.^109^

The second approach is known as albumin-hitchhiking. Since endogenous albumins circulate in the lymphatic system, antigens can be transported to the lymph nodes getting a ride of albumin train if they are able to bind to endogenous albumins.^110,111^ Currently, we know that albumin is a transporter protein for fatty acid molecules, which can capture and bind some lipid molecules.^112^ Using this mechanism, the researchers designed a tumor peptide vaccine with lipophilic tails. Experimental results showed that the vaccine bound albumins and accumulated heavily in lymph nodes, leading to a 30-fold increase in T-cell production and significantly enhanced anti-tumor efficacy after injection in mice.^113^ Another example is 1,2-distearoyl-*sn*-glycero-3-phosphoethanolamine (DSPE), which has been shown to bind endogenous albumin efficiently with high affinity.^114^ Qin et al. constructed a delivery system based on DSPE loaded with OVA antigen peptide and poly-I:C, which allowed the vaccine components to target lymph nodes directly and accumulate in large numbers in lymph nodes, resulting in a strong CD8^+^ T cell response in vivo, leading to a more effective treatment and prolonged median survival in mice.^115^ In addition, endogenous albumin can also bind to some dyes, such as Evans blue dye.^116^ Taking advantage of this feature, Zhu et al. used a derivative of the clinically safe Evans blue to couple to the antigen and found that the vaccine effectively bound to albumin and was delivered to the lymph nodes with the return flow of albumin. The vaccine was found to significantly inhibit the growth of primary or metastatic tumors in mice through in vivo experiments.^117^

#### Promoting antigen cross-presentation

Antigen cross-presentation is the process by which exogenous antigens are presented to CD8^+^ T cells by MHC I molecules. This is an important step for vaccination against viral infections vaccines and cancer vaccines. However, under normal conditions, exogenous antigens are mostly only internalized by APCs alone and presented by MHC II molecules to CD4^+^ T cells, without any cross-presentation can occur.^118,119^ Several delivery systems have been developed to enable antigen cross-presentation by facilitating the escape of antigens from the endosomal or lysosome, which are then loaded by MHC I molecules, thus enabling antigen cross-presentation. These delivery systems achieve this goal in three main ways (Fig. 5).

The first is the proton sponge effect. It occurs when some cationic polymers or lipid delivery systems with protonable amine groups are internalized by APCs and absorb a large number of protons to buffer the acidic environment of the endosomal or lysosome of APCs.^120^ This results in a large influx of chloride ions and water flow from the cytoplasm into the endosomal or lysosome, causing swelling and rupture of the endosome. This process leads to the release of antigens into the cytoplasm, facilitating cross-presentation of antigens by MHC I molecules. For example, under endosomal acidic conditions, polyethyleneimine (PEI) absorbs protons through its protonable amino groups, leading to endosomal swelling and rupture.^121^ A study reported that modification of aluminum hydroxide nanoparticles with PEI significantly increased antigen cross-presentation.^122^ Today, other polycationic polymers and cationic liposomes have also been found to increase antigen cross-presentation through the proton sponge effect.^123–125^

Secondly, some delivery systems destabilize endosomal/lysosomal membranes by fusing or binding to them, thereby releasing antigens into the cytoplasm.^126^ A cationic particulate alum via pickering emulsion (PAPE) particle induced a 3-fold higher number of IFN-γ^+^ T cells compared to the alum group when used to deliver the SARS-CoV-2 RBD vaccine antigen.^127^ This indicates that this particle has a powerful ability to activate cellular immune responses. This is partly due to its ability to bind to the endosomal/lysosomal membrane, which disrupts the stability of the endosomal/lysosomal membrane, leading to the release of antigens into the cytoplasm and their cross-presentation to CD8^+^ T cells.^127^

The third approach is photochemical internalization release technique.^128,129^ This technique provides an emerging technology to route endocytosed material to the cytosol, based on light-induced disruption of endosomal membranes using photosensitizers.^130^ Specifically, the photosensitizers are incorporated into the delivery systems to deliver the antigens together, and subsequently, the photosensitizers are excited to form singlet oxygen after exposure to a specific light source, which causes lipid peroxidation and destruction of endosomal membranes, resulting in the release of antigens into the cytoplasm.^131^ In a study, Ji et al. used arginine and phenylalanine-based polyester amides as raw materials to prepare cationic nanoparticles, which were further subsequently used to form electrostatic complexes with the photosensitizer AlPcS2a and for delivery of antigens. When a light source was applied at 660 nm, it significantly promoted antigen escape from endosomes/lysosomes into the cytoplasm and enhanced CD8^+^ T cell-mediated immune responses.^132^

## Classical adjuvant platforms

Aluminum adjuvants, MF59, AS01, AS03, AS04, and CpG ODN 1018 are classical human vaccine adjuvants. They have been widely approved for use in a wide variety of vaccines and serve to increase vaccine antibody titers and enhance cellular immune responses.^1,133^

### Aluminum adjuvants

**Fig. 6:**
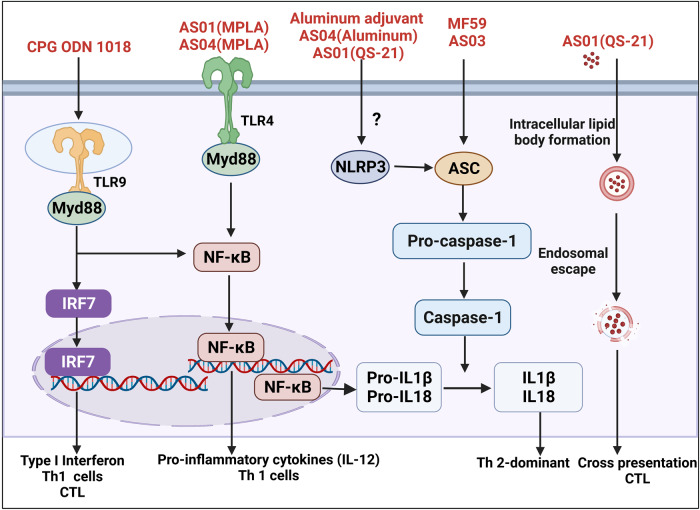
Simplified diagram of the major signaling pathways of classical adjuvants. This figure was created with BioRender (https://biorender.com/)

Aluminum adjuvants are the first adjuvants to be licensed for use in human vaccines. The two commonly used aluminum-based adjuvants in licensed vaccines are aluminum hydroxide and aluminum phosphate. Aluminum adjuvants enhance the production of IgG1 and IgE antibodies by promoting Th2 cell responses. However, the mechanisms of action of aluminum adjuvants is complex and there are still ongoing debates within the academic community.^134^ There are currently two aspects that are well recognized. The first is that aluminum adjuvants act as a delivery system that binds closely to the antigens and sustainedly release of antigens, thus prolonging the bioavailability of the antigen and increasing antigen presentation.^135^ Secondly, aluminum adjuvants can also be used as immunostimulants to induce the production of DAMPs, thereby activating PRRs of innate immune pathways, resulting in the production of cytokines such as IL-1β and Th2-type immune responses.^136,137^ In recent years, several studies have shown that host DNA or uric acid released from host cell death induced by aluminum adjuvant at the injection site acts as endogenous danger signals. These endogenous danger signals can act as DAMPs to induce activation of innate immune pathways.^138–140^ As for the PRR targeted by aluminum adjuvants, some scientists believe it is NLRP3, while some are skeptical (Fig. 6).^136,141,142^ However, this does not deter aluminum adjuvant from becoming the adjuvant of choice for the vaccine industry due to its widely recognized safety and reliability. Aluminum-containing adjuvants are widely used for the prevention and treatment of various diseases, including diphtheria, tetanus, meningitis, and hepatitis B virus (HBV) vaccines, which have been approved by the Food and Drug Administration (FDA).^77^ In addition, aluminum is also a candidate adjuvant for vaccines in new clinical development, such as the SARS-CoV-2 vaccine.^143,144^ However, aluminum adjuvants also have some disadvantages, including difficulties in inducing a strong cellular immune response and the possibility of adverse reactions (erythema, allergic reactions). For these reasons, aluminum adjuvants can be made more effective by improving their formulation or by preparing them as nanoaluminum adjuvants.^127,145–148^

### Emulsion adjuvants

MF59 and AS03 are both classic oil-in-water emulsion adjuvants. MF59 is composed of squalene, Tween 80 and Span 85.^149^ MF59 was licensed as an adjuvant for influenza vaccine in 1997, becoming the first non-aluminum adjuvant approved for use in human vaccines. MF59 emulsion has a dual function of antigen delivery and immune stimulation. MF59 can be used as an emulsion delivery system that, when co-delivered with antigen, it can prolongate antigen interaction with the immune system and increase antigen presentation by slowly releasing antigens in the lymph nodes.^150,151^ This results in more antigenic signals being presented on the surface of APCs, and therefore, the body produces a stronger specific immune response against the antigens.^152^ In addition, MF59 also acts as an immunostimulant. MF59 can target specific PRRs and activate innate immune cells by inducing the production of endogenous danger signals.^153^ The administration of MF59 in muscle activates innate immune cells such as macrophages and DCs, and promotes the production of chemokines such as CC-chemokine ligand 2 (CCL2), CCL4, CCL5, and CXC-chemokine ligand 8 (CXCL8). These chemokines, in turn, recruit more innate immune cells to the injection site to further amplify the immune responses, and promote the migration of these recruited innate immune cells to the draining lymph nodes to activate B and T cells.^1,154^ However, it is worth noting that the exact target PRR of MF59 is still unclear, except that MF59 may function through the NLRP3-independent ASC activation pathway and the TLR-independent MyD88 activation pathway (Fig. 6).^155,156^ In short, vaccination with MF59 adjuvant leads to a Th2-biased immune response and a weak induction of a Th1 response in the body.^56,157^ At present, MF59 has been widely used in a variety of human vaccines and has shown good safety and efficacy.^157–163^

AS03 is another oil-in-water emulsion adjuvant, which consists of alpha-tocopherol, squalence, and Tween 80. The adjuvant effects of AS03 and MF59 are similar. Firstly, it has the function as an antigen delivery system to enhance the presentation of antigen signals on the surface of APCs through slow release. Secondly, AS03 also has immunostimulatory effects because it also contains squalene. And like MF59, AS03 exerts immunostimulatory effects through the NLRP3-independent ASC activation pathway and the TLR-independent MyD88 activation pathway (Fig. 6).^1^ In addition, AS03 has an alpha-tocopherol as an additional immunostimulant component. Studies have shown that alpha-tocopherol is involved in regulating the expression of certain chemokines and cytokines, such as CCL2, CCL3, interleukin 6, and CXCL1, which enhance antigens uptake by APCs and increase the number of innate immune cells recruited to drainage lymph nodes.^164^ Like MF59, in general, AS03 also mainly induces a Th2-biased immune response, with a weak induction of Th1 response.^56^ AS03 was licensed by the European Union for use in influenza vaccines (Pandemrix, TM, GSK) during the 2009 H1N1 pandemic. Subsequently, in 2013, AS03 was licensed by the U.S. FDA for use in the H5N1 avian influenza vaccine and showed satisfactory safety, reactogenicity, and immunogenicity.^165^ AS03 is mainly used in a variety of influenza vaccines to increase the titer and persistence of antibodies.^166,167^ Recently, AS03 has also shown good clinical benefit in the development of the COVID-19 vaccine (NCT04450004, NCT04405908).^168^

### TLR agonist molecule-based adjuvants

AS04 and CpG ODN 1018 are both classical TLR agonist molecule-based adjuvants. AS04 is prepared by aluminum adsorption of TLR4 agonist molecule. Since AS04 contains the aluminum adjuvant, it also has the function of immune stimulation and delivery antigens. However, the immunostimulatory function of AS04 is stronger than that of aluminum adjuvant. This is because AS04 contains a more potent immunostimulatory molecule called monophosphoryl lipid A (MPLA). MPLA, a low-toxicity derivative isolated from LPS, can specifically activate the TLR4 on APCs, leading to the activation of NK-κB and the expression of pro-inflammatory cytokines, resulting in a stronger Th1 cell response (Fig. 6).^169^ In addition, when the vaccine supplemented with AS04 was compared with the vaccine supplemented with aluminum adjuvant only, it was found that the vaccine supplemented with AS04 induced higher levels of antibodies. This proves that TLR4 agonist MPLA plays an important role in AS04.^170–172^ Overall, AS04 produces a stronger antibody response and Th1 cell response than aluminum adjuvants, and induces a more balanced Th1/Th2 immune response.^173^ AS04 is currently approved primarily for use in human papillomavirus (HPV) vaccine (Cervarix) and HBV vaccines (Fendrix) to increase antibody production and activation of antigen-specific T cells.^165,174^

CpG ODN 1018 is a synthetic single-stranded DNA molecule, which has been extensively studied as a TLR agonist.^19^ CpG ODN 1018 specifically activates TLR9, leading to the activation of TRF7, which in turn leads to the production of pro-inflammatory cytokines and type I interferons, ultimately leading to a strong Th1-type cellular response and cytotoxic T cell production.^175^ This results in its ability to produce a better cellular immune response than aluminum adjuvants.^176^ CpG ODN 1018 was initially approved for use in the HBV vaccines.^177^ CpG ODN 1018 is currently being evaluated in clinical trials as a potential vaccine adjuvant for the COVID-19 vaccines (NCT04450004, NCT04405908). Recently, a CpG ODN 1018-adjuvanted COVID-19 vaccine, named SCB-2019, has been evaluated for emergency use.^178^

### Particulate adjuvant system

AS01 is a classical particulate adjuvant system, which is a liposomal adjuvant containing the immunostimulant MPLA and an active ingredient called QS-21 extracted from the bark of *Quillaja Saponaria* (QS).^179^ AS01 has the dual function of antigen presenting and immune stimulator.^180^ The antigen presentation function of AS01 is performed by liposomes. Liposome is a hollow phospholipid bilayer artificial membrane that can be used for encapsulation and delivery of antigens. Liposomes can protect antigens from degradation and prolong their bioavailability, resulting in APCs being able to capture more antigen signals.^180^ The immunostimulatory function of AS01 is contributed by MPLA and QS-21. MPLA activates the innate immune system through TLR4 leading to an increase in Th1-type responses.^180^ QS-21 activates NLRP3 in APCs and subsequently activates caspase 1 to promote the production of active forms of cytokines IL1β, IL18, and IL33 (Fig. 6).^181^ In addition, QS-21 has also been found to promote endosomal escape and promote cross-presentation.^182^ Overall, these two immunostimulant components work synergistically to cause AS01 to induce a Th1-predominant immune response and promote the generation of CTLs.^183^ AS01 is part of licensed malaria and zoster vaccines that promotes antigen-specific antibody production and enhance the cellular immune responses.^6^ Recently, AS01 has also been applied to the development of a novel peptide vaccine against tuberculosis (M72/AS01E) and has shown promise for safety, efficacy, and prevention of tuberculosis in healthy adults (NCT01755598, NCT01755598).^184–186^

In summary, adjuvants are an essential component of vaccines. Classical adjuvants provide a relatively translatable platform for the development of other new vaccines. These adjuvants can be preferentially selected for clinical trials for new vaccines in order to obtain rapid marketing approval. However, these adjuvants have also revealed some problems. For example, they have a weak ability to enhance vaccine immunity, do not provide long-term protective immunity, and are less effective in older populations.^12,13^ Furthermore, most of these approved adjuvants are only capable of inducing antibody responses and have weak ability to induce CD8^+^ T cell-mediated cellular immunity, which is critical for vaccines against viral infectious diseases and cancer vaccines. Last but not least, the action mechanisms of most of the licensed adjuvants remain largely unclear, which may lead to inappropriate use of these adjuvants and uncontrollable side effects. Therefore, novel adjuvants are needed to remedy these problems.

## Adjuvant platforms under investigation

Due to the prominent role of adjuvants in vaccines, adjuvant science has gone through rapid development in recent years. Some of the immunostimulants and delivery systems under investigation show good adjuvant efficacy. Next, we will summarize and describe them.

### Immunostimulant platforms under investigation

In recent years, with the intensive study of adjuvants, some of the immunostimulants under investigation have shown good adjuvant efficacy. In this section, we will summarize and describe the mechanisms of action, properties, and application progress of these immunostimulants under investigation.

#### Synthetic double-stranded RNAs (dsRNAs)

Synthetic double-stranded RNAs (dsRNAs) can target and activate TLR3 and MDA5 on APCs, leading to the production of pro-inflammatory cytokines such as IL-12 and type I interferons, and promoting the production of strong Th1-biased immune responses and CTLs. Poly-I:C and its modified variant Poly-ICLC are the most studied synthetic dsRNA immunostimulants.^187–189^ In vitro, poly-I:C and poly-ICLC induce maturation of human peripheral blood monocyte-derived DCs, leading to secretion of IFN-β and the pro-inflammatory cytokines IL-6 and IL-12, resulting in cross-presentation of exogenous antigens with CD8^+^ T cells and triggering a Th1-polarized immune response.^190,191^ Both preclinical and clinical studies have shown that poly-I:C and poly-ICLC are promising adjuvants that enhance antibody production and CD8^+^ T cell immune responses.^192,193^ A test in humans showed that combining poly-ICLC with a DC-targeted vaccine induced an innate immune response similar to that of a live virus vaccine, further emphasizing the effectiveness of poly-I:C/poly-ICLC as vaccine adjuvants.^194^ In addition, we note that poly-I:C and poly-ICLC are used clinically in peptide vaccines, DC vaccines, and whole cell vaccines, and are primarily indicated for a wide variety of cancer vaccines (Table 1). The addition of poly-I:C or poly-ICLC to cancer vaccines enhances vaccine-induced anti-tumor T cell and NK cell responses, thereby contributing to tumor regression or eradication.^195^ However, it is worth noting that poly-I:C/poly-ICLC may have side effects such as dose-dependent systemic fever and coagulation abnormalities, in addition to its function of triggering a powerful immune response.^196–198^ Therefore, a rational delivery system should be considered to protect it from degradation and to target more poly-I:C/poly-ICLC to APCs, in order to ensure the lowest possible dose to activate APCs and reduce side effects.^16^

**Table 1:** Clinical trials of immunostimulant platforms under investigation (2016–2023)

Platform type	Platform name	Mechanisms and effects	Antigen	Tested diseases	NCT number	Phases
**Immunostimulant platforms of infectious disease vaccines**						
Synthetic dsRNA	Poly-ICLC	Activating TLR3 and MDA5 to enhance antigen-specific CD4^+^ T and CD8^+^ T cellular immune response production.	Gag p24 recombinant protein	HIV infections	NCT01127464	I
GLA and derivatives	GLA-SE	Activating TLR4 to enhance antigen-specific antibodies and Th1-type cellular immune response production.	ID93 recombinant protein	Tuberculosis	NCT01927159	I
			ID93 recombinant protein	Tuberculosis	NCT02465216	II
			PAMVAC protein	Malaria	NCT02658253	I
			PAMVAC protein	Malaria	NCT02647489	I
			H5 VLP recombinant protein	Influenza	NCT01991561	II
			CH505TF gp120 protein	HIV infections	NCT04607408	I
			LEISH-F3 recombinant protein	Visceral leishmaniasis	NCT02071758	I
			Sm14 protein	Schistosomiasis	NCT03799510	II
	GLA-AF	Activating TLR4 to enhance antigen-specific antibodies and Th1-type cellular immune response production.	Na-APR-1 (M74) recombinant protein	Hookworm infection	NCT01717950	I
			CN54rgp140 DNA	HIV infections	NCT01922284	I
Imidazoquinolines	Imiquimod	Activating TLR 7/8, promoting the production of pro-inflammatory cytokines and type I interferons, which will lead to Th1 cell and CTLs production.	Recombinant protein	HBV	NCT02621112	II/III
			Recombinant protein	HPV	NCT02689726	I
			Pre-S1/Pre-S2/S recombinant protein	Hepatitis B	NCT03307902	II/III
	3M-052	Activating TLR 7/8, promoting the production of pro-inflammatory cytokines and type I interferons, which will lead to Th1 cell and CTLs production.	CH505 TF trimer recombinant protein	HIV infection	NCT04915768	I
			Inactivated SARS-CoV-2 virus	COVID-19	NCT04641481	II
CpG ODNs	CpG ODN 1018	Activating TLR9 to induce Th1-type response and the production of CTLs.	COVID-19 VLP recombinant protein	COVID-19	NCT04450004	I
			SARS-CoV-2 trimeric S protein	COVID-19	NCT04405908	I
			SARS-CoV-2 recombinant protein	COVID-19	NCT05228613	I
			Hepatitis B Virus recombinant protein	Hepatitis B	NCT00426712	I
			rF1V recombinant protein	Plague	NCT05506969	II
	CpG ODN 7909	Activating TLR9 to induce Th1-type response and the production of CTLs.	AMA1recombinant protein	Malaria	NCT00427167	I
			AMA1 and MSP1 recombinant protein	Malaria	NCT00889616	I
	IC31	(1) Activating TLR9 to induce Th1-type response and the production of CTLs; (2) Sustained-release antigens to enhance antigen presentations.	Recombinant protein	Tuberculosis	NCT03512249	II
			Synthetic peptide	Hepatitis B	NCT02496897	I
			Recombinant protein	Tuberculosis	NCT02075203	II
CDNs	c-di-AMP	Activating cGAS-STING pathway to induce strong Th1-type cellular response and CTLs production.	PANHPVAX recombinant protein	HPV	NCT05208710	I
**Immunostimulant platforms of tumor vaccines**						
Synthetic dsRNA	Poly-ICLC	Activating TLR3 and MDA5 to enhance antigen-specific CD4^+^ T and CD8^+^ T cellular immune response production.	MUC1 peptide	Colorectal adenoma	NCT02134925	II
			MUC1 peptide	Advanced colorectal adenoma	NCT00773097	II
			IMA 950 peptide	Glioblastoma	NCT01920191	I/II
			MUC1 peptide	Non-small cell lung cancer	NCT01720836	I/II
			HLA-A2 restricted glioma peptide	Pediatric gliomas	NCT01130077	I
			MUC1 peptide	Triple-negative breast cancer	NCT00986609	I
			Tumor cell lysate	Malignant glioma	NCT01204684	II
			Glioma-associated peptide	Gliomas	NCT00874861	I
			Tumor cell lysate	Ovarian cancer	NCT02452775	I
GLA and derivatives	GLA-SE	Activating TLR4 to enhance antigen-specific antibodies and Th1-type cellular immune response production.	MART-1 peptide	Melanoma	NCT02320305	I
			NY-ESO-1 protein	Tumor	NCT02015416	I
Imidazoquinolines	Imiquimod	Activating TLR 7/8, promoting the production of pro-inflammatory cytokines and type I interferons, which will lead to Th1 cell and CTLs production.	NY-ESO-1 protein	Malignant melanoma	NCT00142454	I
			MAGE-3A1 peptide, or the NA17.A2 peptide	Metastatic melanoma	NCT01191034	I/II
			Peptide	Leukemia, Chronic Lymphatic	NCT02802943	II
			Tumor cell lysate	Malignant glioma	NCT01204684	II
	Resiquimod	Activating TLR 7/8, promoting the production of pro-inflammatory cytokines and type I interferons, which will lead to Th1 cell and CTLs production.	NY-ESO-1 protein	Tumors	NCT00821652	I
			Gp100 and MAGE-3 peptide	Melanoma	NCT00960752	II
			Peptide	Melanoma	NCT02126579	I/II
			MART-1 peptide	Melanoma	NCT01748747	I
CpG ODNs	CpG ODN 7909	Activating TLR9 to induce Th-type response and the production of CTLs.	MAGE-3 A1 peptide	Melanoma	NCT00145145	I/II
			NY-ESO-1 protein	Tumors	NCT00299728	I

#### GLA and derivatives

Glucopyranosyl lipid A (GLA) is a synthetic LPS mimic that is an alternative to MPLA. GLA can induce Th1-type immune responses in vivo by activating TLR4 on the APCs.^199^ To enhance the immune effect of GLA, it is usually prepared as a formulation for use. GLA is commonly prepared as glucopyranosyl lipid A-aqueous nanosuspension (GLA-AF), glucopyranosyl lipid A-stable emulsion (GLA-SE), glucopyranosyl lipid A-liposome (GLA-LS) and glucopyranosyl lipid A-aluminum hydroxide (GLA-alum).^169^ Among them, GLA-SE is the most studied in preclinical and clinical studies. When Clegg et al. used GLA-SE as an adjuvant for the H5N1 subunit vaccine, they found that it induced the production of antigen-specific T1-type CD4^+^ T cells and higher titers of Th1-type antibodies, and protected mice and ferrets from H5N1 virus challenge.^200^ In non-human primates, GLA-SE induces more Th1-type cytokines when used as an influenza vaccine adjuvant and induces the production of neutralizing antibodies against multiple influenza virus variants, greatly improving the protective immune effect of the vaccine.^201^ In addition to improved humoral immune responses to the vaccines, in a phase II clinical trial, when used GLA-SE as an adjuvant for the H5N1 influenza vaccine, showed a dose saving effect, which is particularly beneficial in pandemic influenza situations (NCT01991561).^202^ In several clinical trials, when GLA-SE was used as an adjuvant for TB vaccine and tested against ID93 recombinant protein, the vaccine was found to induce high titer antigen-specific antibody production and CD4^+^ T responses, demonstrating good safety and immunogenicity in vaccinated populations (NCT01599897, NCT02465216, NCT02508376, NCT01927159).^203,204^ In addition, GLA-SE has been clinically evaluated as a vaccine adjuvant in malaria vaccines, tumor vaccines, HIV vaccines, visceral leishmaniasis vaccines, and schistosomiasis vaccines (Table 1). These clinical trials seem to suggest that GLA-SE is an effective and safe vaccine adjuvant with potential as a next-generation vaccine adjuvant. GLA-AF is another well-studied product of the GLA family. GLA-AF has also been evaluated in preclinical and clinical tests as an adjuvant candidate for several vaccines (NCT01717950, NCT01922284).^205,206^

#### Imidazoquinolines

Imidazoquinolines are able to activate TLR 7/8, leading to activation of NF-κB and IRF7, promoting the production of pro-inflammatory cytokines and type I interferons, which will lead to strong Th1 cell and CTLs production.^207^ Imiquimod (R837), resiquimod (R848), and 3M-052 (also known as telratolimod) are the most studied imidazoquinolines as adjuvants in preclinical and clinical settings (Table 1). Substantial evidence suggests that R837 exhibits strong adjuvant action in melanoma, HPV, breast cancer, and T-cell lymphoma vaccines.^208–211^ Viral vaccines and tumor vaccines coupled with R848 have shown to induce stronger humoral responses and CD8^+^ T cell immune responses.^212,213^

3M-052 is an imidazoquinoline compound with a structure similar to that of R848. The 18-C fatty acyl chain of 3M-052 confers enhanced hydrophobicity to the compound, thereby improving bioavailability at the immune site and reducing the probability of systemic transmission.^214^ In addition, this lipidation makes it easier to incorporate 3M-052 into lipid-based formulations, such as emulsions or liposomes. HIV vaccines formulated with 3M-052 encapsulated in PLGA nanoparticles or alum have been shown to induce high and sustained levels of antibody responses and T-cell responses.^215–218^ An HIV vaccine based on alum-3M-052 adjuvant has been studied in a human phase 1 clinical trial (Table 1, NCT04915768). In addition, 3M-052 is also used in an inactivated SARS-CoV-2 vaccine. Interim results from a phase 3 trial in India showed an overall estimated vaccine efficacy of 77.8%, which indicated the vaccine was well tolerated (Table 1, NCT04641481) (COVAXIN, BBV152).^219^ COVAXIN is currently approved by the World Health Organization (WHO) for emergency use.

The use of these imidazoquinolines as vaccine adjuvants enhances immune efficacy, but suffers from several shortcomings. Specifically, they often diffuse systemically from the injection site, thereby moving away from the antigens, reducing efficacy, and inducing systemic side effects.^207^ Therefore, these imidazoquinolines need to be coupled with synthetic polymeric scaffolds, nanogels, lipid-based nanoparticles or other delivery materials to enhance their immune effects and reduce their side effects.^220^

#### CPG ODNs

CPG ODNs are synthetic single-stranded DNA molecules, which have been extensively studied as TLR9 agonists.^221^ Among them, the most commonly used as vaccine adjuvants are CpG ODN 1018, CpG ODN 7909, and IC31. They can lead to transcriptional activation of TRF7, which in turn leads to the production of cytokine IL-12 and type I interferons, ultimately inducing a strong Th1-type cell response and CTLs production. CpG ODN 1018 is the most studied CPG ODNs in preclinical and clinical trials, and it has been described in the previous section.

In addition to CpG ODN 1018, CpG ODN 7909 is the most studied CpG ODN. When CPG ODN 7909 is added to the HBV vaccine, CPG ODN 7909 induces a more significant and rapid HBsAg-specific humoral response in healthy populations.^222^ CPG ODN 7909 can also improve the response rate of HIV-positive patients to vaccination, demonstrating the promising potential of vaccines formulated with CPG ODN 7909 to improve vaccine response rates in immunocompromised individuals.^223,224^ A randomized, double-blind controlled trial tested the immunostimulatory effects of CPG ODN 7909 as an adjuvant for the HBV vaccine (Engerix-B). The results of the trial showed that the addition of CPG ODN 7909 achieved rapid, higher and sustained seroprotection and increased HBV-specific Th responses compared to Engerix-B alone.^225^ In addition to its use with HBV, CPG ODN 7909 is also used in malaria vaccines. A phase I clinical trial of BSAM2, a protein-based malaria vaccine, showed a dose-sparing effect of adding CpG ODN 7909 (Table 1, NCT00889616).^226^ This feature is particularly valuable in resource-poor environments.

IC31 is another agonist of TLR9, which contains both CPG ODN and a positively charged antimicrobial peptide component. It signals cellular and humoral immune responses via a TLR9/MyD88-dependent pathway. In addition, the stable complex formed by the ionic and hydrophobic interactions between the two components of IC31 can form an antigen reservoir at the injection site, providing a slow release of antigen and prolonging the bioavailability of antigen.^227^ IC31 can increase antigen presentation by APCs and significantly improve the immune effect of CPG ODN. Mouse-based studies have shown that IC31 contributes to the induction of potent antigen-specific CTLs and strong antigen-specific humoral responses.^228^ IC31 has been extensively evaluated in preclinical and clinical trials as a candidate adjuvant for a variety of subunit vaccines (NCT03512249, NCT02496897, NCT02075203).^229–234^ The results indicate that IC31 is an effective vaccine adjuvant with potential for clinical translation.

#### Cyclic dinucleotides (CDNs)

CDNs can target and activate the cGAS-STING pathway, leading to the activation of IRF3 and NF-κB, inducing the production of type I interferons and proinflammatory cytokines.^235^ The type I interferons selectively stimulates antigen cross-presentation and mobilizes CD8 ^+^ T cells. As a result, CDNs generate strong Th1-type cell responses and CTLs responses. The CDNs include natural CDNs and synthetic CDNs. Natural CDNs include 2’,3’-cGAMP, 3’,3’-cGAMP, c-di-GMP, and c-di-AMP, which are second messengers of bacteria and mammals with strong immunomodulatory functions.^236^ Adjuvants based on natural CDNs have been evaluated in a variety of vaccines and have shown promising results. For example, when 2’,3’-cGAMP is encapsulated in pulmonary surfactan-mimetic liposomes, it enhances H1N1 influenza vaccine-induced humoral and CD8^+^ T cell responses and induces long-term cross-protective immune responses against heterotypic viruses in mice and ferrets.^237^ Junkins et al. encapsulated 3’3’-cGAMP in acid-sensitive acetal dextran (Ace-DEX) polymer particles and administered them to mice together with an influenza vaccine. The test results showed 60 to 600 times higher in antibody titers than those of the regular alum vaccine group and caused no observable toxic effects.^238^ Lin et al. encapsulated c-di-GMP in PLGA and externally coupled the MERS vaccine antigen to form a nanovaccine. This vaccine not only triggered effective neutralizing antibodies and antigen-specific T-cell responses, but also preferentially targeted draining lymph nodes, generating local immunity and reducing systemic responses.^239^ When Ebensen used c-di-AMP as a mucosal adjuvant for the H5N1 influenza vaccine, it was found to effectively induce protective immunity against H5N1 influenza in mice.^240^ Phase I clinical trial of c-di-AMP as HPV vaccine adjuvant is currently underway (Table 1, NCT05208710).

Due to the short half-life and low uptake efficiency of natural CDNs by APCs, some synthetic CDNs with chemical modifications have emerged to enhance their immune effects, such as ADU-S100, MK-1454, BMS-986301, SB-11285, IMSA-101. However, there are no specific examples of the use of these synthetic CDNs as vaccine adjuvants. It is worth noting that, since these synthetic CDNs have shown the ability to significantly enhance the body’s immune responses in preclinical and clinical trials, we believe that the desire to use these synthetic CDNs as vaccine adjuvants will soon be realized (NCT03172936, NCT03010176, NCT03010176, NCT04096638, NCT04020185).^241,242^

Furthermore, it is noteworthy that with the advent of nanotechnology, nanoparticle-based delivery systems such as liposomes, emulsions, virus-like particles (VLPs) and biodegradable polymers, have received significant attention. They can continuously release the cargo and improve the bioavailability of the cargo. Therefore, the use of nanoparticles to encapsulate CDNs has been seen as a strategy to improve the utilization efficiency of CDNs in vivo.^237,243,244^

#### Metabolic adjuvants

In recent years, some small molecule modulators targeting metabolic pathways have been found to be novel vaccine adjuvants. The mevalonate pathway is the core metabolic pathway of various cellular processes, including cholesterol biosynthesis and protein post-translational prenylation.^245^ Researchers have found that lipophilic statins and rationally designed bisphosphonates can inhibit the formation of the downstream metabolite geranylgeranyl pyrophosphate (GGPP) by targeting the enzymes in the mevalonate pathway.^246^ This result leads to blocked geranylgeranylation of small GTPases (e.g., Rab5) in APCs, which slows down the transport of antigens from the endosomes to the lysosomes and prevents rapid degradation of antigens.^247^ This prolongs antigen retention so that antigen presentation is enhanced. These small-molecule inhibitors targeting mevalonate pathways have shown adjuvant effects in increasing antibody titers and enhancing cellular immune responses in both mice and cynomolgus monkeys.^246^ In addition, recent studies have found that the mammalian target of rapamycin (mTOR) complex, a central metabolic regulator, plays an important role in regulating immune cells. First of all, mTOR complex can regulate the secretion of type I interferons in plasmacytoid DCs.^248^ Inhibition of mTOR complex will severely reduce the interferon α produced. Moreover, the mTOR complex is an essential regulator of effector T cell expansion and germinal center B cell response production.^249^ In addition to mTOR, the amino acid sensor general control nonderepressible 2 (GCN2) also plays a role in regulating immune cells such as DCs. Studies have shown that activation of GCN2 leads to enhanced autophagy of DCs, which enhances the antigen presentation.^250^ These results suggest that enzymes or regulators of cellular metabolic pathways may be potential targets for the design and development of novel adjuvants.

#### Manganese adjuvants and derivatives

Recently, Manganese (Mn) and its derivatives have been found to have potential adjuvant activity. They act as activators of cGAS, directly activating cGAS and inducing a noncanonical catalytic synthesis of 2’3’-cGAMP.^251^ In this way, manganese activates the innate immune pathway of cGAS-STING, induces the production of type I interferons, enhances antigen presentation and cross-presentation, promotes the production of antibodies and increases the production of CD8^+^ T cell immune responses.^252,253^ It is reported that Mn^2+^ induced mouse bone marrow-derived dendritic cells to produce a large amount of IFNβ and IFNα, and led to significant up-regulation of costimulatory molecules (e.g., CD80 and CD86) and chemokines (e.g., CCL2 and CCL3) in vitro experiments.^254^ This suggests that Mn^2+^ has adjuvant activity to induce DCs maturation and enhance antigen presentation. To further verify its adjuvant activity, the researchers injected a solution containing Mn^2+^ with the antigen into mice and found that it led to an increase in antibody titers. To prevent Mn^2+^ from aggregating in solution and losing adjuvant activity, researchers have developed a series of Mn^2+^-based nanoadjuvants to stabilize the adjuvant activity of Mn^2+^.^254,255^ For example, nanoscale manganese jelly (MnJ), which not only has the ability to activate the cGAS-STING pathway as an immunostimulant to induce the production of type I interferons, but also serves as a delivery system to carry the antigen, resulting in strong humoral and cellular responses.^254–256^ When administered intranasally, MnJ also acts as a mucosal adjuvant to induce high levels of IgA antibodies. Wang et al. developed another manganese adjuvant called MnARK. They found that even at a 5-fold lower antigen dose and a reduced number of injections, mice vaccinated with the RBD vaccine containing MnARK adjuvant showed greater neutralization of infection with pseudovirus (around 270-fold) and live coronavirus (8-fold) compared to mice vaccinated with the RBD vaccine containing aluminum adjuvant.^257^ Sun et al. used the chemical engineering strategies to fabricate a nano-manganese adjuvant based on Mn^2+^ called nanoMn. The coronavirus vaccine with nanoMn as an adjuvant induced a strong CD8^+^ T cell immune response and showed good safety in vivo.^258^ In addition, some other nano-adjuvants based on Mn^2+^ have also shown the effect of activating the cGAS-STING pathway and improving the immune responses of vaccines.^259,260^ These results suggest that manganese has great potential as a target for the development of novel vaccine adjuvants.

A number of immunostimulants with different properties have been evaluated in preclinical and clinical trials. Overall, immunostimulants that target TLRs remain the most popular in preclinical and clinical studies. On the other hand, there is an increasing trend to combine multiple PRRs agonists and formulate these agonists into oil-in-water emulsions or load them using other delivery systems to enhance their targeting of APCs (NCT02126579, NCT01585350, NCT01008527, NCT02126579).

### Delivery system platforms under investigation

In recent years, with the development of engineering materials science, a variety of vaccine delivery systems based on engineering materials have been developed. Novel water-in-oil nanoemulsions, LNPs, polymer nanoparticles, VLPs, caged protein nanoparticles and inorganic nanocarriers are some of the important delivery system platforms. Different types of delivery systems have different mechanisms of action and physicochemical properties, which correspondingly affect the efficacy of vaccination. Here, we summarize and describe the mechanisms, properties, and applications of these delivery system platforms.

#### Novel water-in-oil nanoemulsion

Montanide ISA 51 and Montanide ISA 720 are two novel water-in-oil emulsion delivery systems being tested in clinical trials.^77^ Montanide ISA 51 is emulsified with mineral oil and mannide monooleate, while Montanide ISA 720 is emulsified with non-mineral oil and mannide monooleate. In addition, they have different oil-to-water ratios.^261^ The mechanisms of action of these two adjuvants are the formation of an antigen depot and the slow release of antigen to prolong the bioavailability of antigens.^262^ Both preclinical and clinical test results show that Montanide ISA 51 and Montanide ISA 720 adjuvants enhance serum antibodies production and CTLs production.^263–265^ Currently, Montanide ISA 51 and Montanide ISA 720 are being evaluated in clinical trials as adjuvants for influenza, malaria, melanoma, and other cancer vaccines (Table 2). In addition, Montanide ISA 51 is licensed for therapeutic lung cancer vaccine in Cuba.^77^ We note that Montanide ISA 51 and Montanide ISA 720 are often used as emulsified immunostimulant adjuvants to enhance the potency of immunostimulants (NCT00199836, NCT01008527, NCT01585350).

**Table 2:** Clinical trials of delivery system platforms under investigation (2016–2023)

Platform type	Platform name	Mechanisms and effects	Antigen	Tested diseases	NCT number	Phases
**Delivery system platforms of infectious disease vaccines**						
W/O emulsion	Montanide ISA 51	(1) Forming a depot at the injection site; (2) Sustained-release antigens to enhance antigen presentations.	PPfs5 and ScPvs20 recombinant protein	Malaria	NCT00295581	I
			FLU-v peptide	Influenza	NCT02962908	II
			CS protein	Malaria	NCT02083068	II
			CS protein	Malaria	NCT04739917	II
	Montanide ISA 720	(1) Forming a depot at the injection site; (2) Sustained-release antigens to enhance antigen presentations.	PfCP2.9 recombinant protein	Malaria	NCT00284973	I
LNPs	LNP	(1) Providing effective cargo protection for antigens and prolonging antigen bioavailabilities; (2) Increasing the particle size of antigens to target antigens to APCs and lymph nodes; (3) Promoting antigens escape from endosomes by membrane fusion and leading to CD8^+^ T cell immune response.	mRNA	Influenza	NCT03076385	I
			mRNA	Zika	NCT03014089	I
			mRNA	Rabies	NCT03713086	I
			mRNA	RSV	NCT04528719	I
			mRNA	Chikungunya	NCT03829384	I
			mRNA	Cytomegalovirus	NCT03382405	I
			ARCT-021 mRNA	COVID-19	NCT04480957	I/II
			mRNA	Influenza	NCT03345043	III
			mRNA	COVID-19	NCT04470427	III
			mRNA	COVID-19	NCT04368728	III
			mRNA	COVID-19	NCT04860258	III
VLPs	VLP	(1) Trafficking antigen to lymph nodes; (2) Displaying multivalent antigens; (3) Cross-linking with BCRs to strongly activate B cells.	H7N9 VLP protein	H7N9 Influenza	NCT01897701	I
			COVID-19 VLP protein	COVID-19	NCT04450004	I
			A Trivalent VLP protein	Encephalitis	NCT03879603	I
			COVID-19 VLP protein	COVID-19	NCT05040789	III
			PXVX0317 Chikungunya VLP protein	Chikungunya Virus	NCT05072080	III
Caged protein nanoparticles	Ferritin	(1) Trafficking antigens to lymph nodes; (2) Displaying multivalent antigens; (3) Cross-linking with BCRs to strongly activate B cells.	Hemagglutinin protein	Influenza	NCT03186781	I
			Hemagglutinin protein	Influenza	NCT04579250	I
			Hemagglutinin protein	Influenza	NCT03814720	I
			EBV gp350 protein	Epstein-Barr virus infection	NCT04645147	I
			SARS-CoV-2 recombinant spike protein	COVID-19	NCT04784767	I
Inorganic nanocarriers	Gold nanoparticle	Forming nanovaccines with appropriate particle size to target antigens to APCs and lymph nodes.	Peptide	Dengue	NCT04935801	I
			Peptide	COVID-19	NCT05113862	I
**Delivery system platforms of tumor vaccines**						
W/O emulsion	Montanide ISA 51	(1) Forming a depot at the injection site; (2) Sustained-release antigens to enhance antigen presentations.	NY-ESO-1 peptide	Cancer	NCT00199836	I
			Mixed TAA peptide	Central nervous system tumors	NCT00935545	I
			P10s‐PADRE peptide	Breast cancer	NCT01390064	I
			Gp100 peptide	Melanoma	NCT00003274	II
			HLA-A2-restricted peptides	Melanoma	NCT00145158	I/II
			Peptide	Prostate cancer	NCT02452307	I/II
	Montanide ISA 720	(1) Forming a depot at the injection site; (2) Sustained-release antigens to enhance antigen presentations.	NY-ESO-1 protein	NY-ESO-1-expressing tumors	NCT00819806	I
LNPs	LNP	(1) Providing effective cargo protection for antigens and prolonging antigen bioavailabilities; (2) Increasing the particle size of antigens to target antigens to APCs and lymph nodes; (3) Promoting antigens to escape from endosomes by membrane fusion and leading to CD8^+^ T cell immune response.	KRAS mRNA	Tumor	NCT03948763	I
			mRNA	Melanoma	NCT02410733	I
			mRNA	Triple-negative breast cancer	NCT02316457	I
			mRNA	Gastrointestinal cancer	NCT03480152	I/II
			mRNA	Melanoma	NCT03897881	II
			mRNA	Melanoma	NCT03815058	II
Polymeric particles	PLGA	Sustained-release antigens to enhance antigen presentations.	Autologous melanoma cell lysate	Melanoma	NCT01753089	I
			NY-ESO-1 peptide	Advanced solid tumor	NCT04751786	I

#### Lipid nanoparticles (LNPs)

LNPs are multifunctional, non-viral, nanoscale lipid vesicle delivery systems that consist of ionizable lipids, phospholipids, cholesterol, and polyethylene glycol modified lipids.^266^ Ionizable lipids are the main components of the LNPs. Phospholipids and cholesterol contribute to the structural integrity of LNPs, and polyethylene glycol modification helps to maintain the stability of LNPs. The mechanisms of action of LNPs mainly include (1) providing effective cargo protection for antigens and prolonging their bioavailability; (2) increasing the particle size of antigens to target APCs and promote their uptake by APCs; (3) promoting antigen escape from endosomes via membrane fusion, leading to CD8^+^ T cell immune response.^267^ After years of research, LNPs have been used in the delivery of various vaccines and have shown to strongly enhance both humoral and cellular immune responses. Alameh and his colleagues found that LNPs enhanced humoral immune responses to mRNA and protein subunit vaccines by inducing the proliferation of powerful T follicular helper cells, germinal center B cells, long-lived plasma cells, and memory B cells.^268^ Oberli and his colleagues induced activation of CD8^+^ T cells when using LNPs to deliver mRNA vaccines for the tumor-associated antigens gp100 and TRP2 to treat B16F10 melanoma, which led to tumor shrinkage and prolonged overall survival in mice.^266^ In recent years, LNPs have been extensively tested as a promising mRNA vaccine delivery system in clinical trials for COVID-19 vaccines, tumor vaccines, influenza vaccines, etc (Table 2). In 2020, Pfizer’s BNT162b2 vaccine (known as Comirnaty) and Moderna’s mRNA-1273 vaccine (known as Spikevax), both of which deliver mRNA antigens with LNPs, were approved for emergency marketing. These two mRNA vaccines have made a significant contribution to the fight against COVID-19.^269,270^

#### Polymer particles

Polymer particles are generally divided into natural polymer and synthetic polymer particles. Usually, the mechanism by which they function is through the slow release of antigens. The most common natural polymer used in vaccine delivery is chitosan. Chitosan has a high cationic charge and bioadhesive properties.^271^ Therefore, chitosan can form a tight complex with anionic nucleic acid through electrostatic interaction.^272,273^ Furthermore, the bioadhesive properties of chitosan allow it to remain in contact with mucosal surfaces for a long time, prompting continuous stimulation of immune cells by antigens. This indicates that chitosan may be a promising delivery system for nucleic acid vaccines and mucosal vaccines. Sawaengsak et al. prepared a nanoparticle vaccine by cross-linking chitosan with sodium tripolyphosphate to encapsulate inactivated influenza virus antigens and found that mice that received 2 doses of this vaccine intranasally were able to produce more antigen-specific antibodies and IFNγ^+^ T cells. This resulted in 100% protection against influenza virus attack in vaccinated mice.^274^ Recently, chitosan has also been tested as a delivery system for Neocrown DNA vaccines. After intranasal immunization, high levels of neutralizing antibodies were detected in mice, and various Corona Virus and their mutant pseudoviruses were effectively neutralized.^275^ In addition, chitosan has also recently been found to induce type I interferons production for CD8^+^ T cell immunity by activating the DNA sensor cGAS-STING pathway.^276^

Synthetic polymer particles typically have higher reproducibility and a more controlled rate of slow release than natural polymers.^277^ PLGA, a synthetic polymeric particle material, is now widely used as a carrier for vaccine delivery. In preclinical studies, Kim et al. encapsulated vaccine components (including antigens and immunostimulants) in PLGA to increase uptake and presentation of antigen by DCs, which allowed the immunostimulant to target DCs in lymph nodes more than systemic spread.^278^ Koerner et al. designed a PLGA particle that encapsulated antigens and dsRNA adjuvants. The particle vaccine was found to exhibit an ideal release curve, increased the number of targeted lymph nodes, and was effectively phagocytosed and presented by DCs. Finally, an effective and lasting anti-cancer immune response was produced.^279^ PLGA is also used to deliver other cancer vaccines and HBV vaccines and to enhance their immunotherapeutic effects.^280–283^ At present, PLGA particles have been clinically tested as delivery systems for cancer vaccines (NCT01753089, NCT04751786).^284^ It is worth noting that PLGA has been approved by the FDA for drug delivery. Therefore, we believe that a PLGA-based vaccine delivery system will be approved for human use in the near future.^285,286^

#### Virus-like particles (VLPs)

VLPs are polymeric particles with a fixed shape derived from the coat proteins of viral capsids and formed by the self-assembly of protein monomers.^287^ VLPs’ excellent particle sizes and geometry make it an effective platform for delivery of vaccine antigens.^288,289^ VLPs are typically between 20 and 100 nm in diameter and therefore readily enter lymphatic vessels and target lymph nodes for uptake by specialized APCs.^11^ In addition, due to their highly repetitive and rigid structure, VLPs can display multivalent antigenic epitopes on their surface and therefore can extensively cross-link BCRs, thereby stimulating B cells and inducing a robust and long-lasting antibody response.^290^ Currently, VLP-based vaccines have been successful and are already widely available in the market, such as Cervarix^®^ and Gardasil^®^ for HPV, Sci-B-Vac™ for hepatitis virus, and RTS, S VLP vaccine.^291^ VLPs can be used to deliver not only endogenous viral antigens, but also heterologous antigens modified on VLPs by chemical coupling or gene fusion. For example, a breast cancer VLP vaccine that attaches HER2 antigenic epitopes to 30 nm icosahedral cowpea mosaic virus (CPMV) could effectively induce antigen-specific responses and tumor protection in a mouse model.^292^ In addition, the tumor-associated carbohydrate antigen MUC1 can be covalently linked to Qβ, a self-assembled icosahedral shell VLP with 25 nm. This MUC1 VLP vaccine induces higher levels of specific antibodies and prolongs the survival of tumor-bearing mice.^293^ Several VLP-based vaccines have successfully entered clinical trials, including the chikungunya vaccine, influenza vaccine, Neocon vaccine, cancer, encephalitis and other vaccines (Table 2).^294^ Notably, due to the highly ordered and repetitive spatial structure of VLPs, which is highly conducive to cross-linking BCRs, the VLPs vaccines can strongly activate B cells even in the absence of T helper (Th) cells.^288^

#### Caged protein nanoparticles

Caged protein nanoparticles typically consist of a series of repeating motifs that allow antigenic epitopes or antigens to be incorporated into their subunit structures, so that these incorporated antigenic epitopes or antigens are displayed on the surface of the assembled particles.^295^ Similar to VLPs, caged protein nanoparticles have properties such as highly ordered and repetitive spatial structure and optimal size for lymph node transit. However, unlike VLPs, caged protein nanoparticles are of non-viral origin. Caged protein nanoparticles such as heat shock proteins,^296^ protein vault,^297,298^ and ferritin protein^299^ have been extensively studied as delivery platforms for vaccines in preclinical and clinical settings. Among the above protein nanoparticles, ferritin is the most frequently applied. Ferritin is a protein with an octahedral symmetry structure formed by the self-assembly of 24 subunits, each of which consists of four alpha-helix bundles.^300^ Kanekiyo et al. constructed a nano-influenza vaccine with an antibody titer more than 10 times higher than licensed inactivated vaccines by genetically fusing the hemagglutinin antigen of the H1N1 influenza virus with a ferritin of Helicobacter pylori origin, and inserting the hemagglutinin antigen into the ferritin subunit and displaying it on its surface.^301^ Promising results obtained with this design have helped develop three vaccines against other influenza subtypes, which are now in phase 1 clinical trials (NCT03186781, NCT03814720, NCT04579250). In the above three phase 1 clinical trial, the trial NCT03186781 has been completed. The results show that the influenza vaccine H2HA-ferritin, whether as a stand-alone or booster regimen, is safe and well tolerated, and can produce a broadly neutralizing antibody response.^302^ Recently, Zhang and his colleagues developed a ferritin-based SARS-CoV-2 nanoparticle vaccine that elicited an effective protective immune response.^303^ In addition, the HBV vaccine based on ferritin nanoparticles can deliver HBV antigen preS1 to specific APCs, activate a strong immune response, and induce a high level of sustained antibody responses.^86^ In addition, ferritin nanoparticles can effectively deliver tumor-specific antigens to lymph nodes, resulting in specific cytotoxic CD8^+^ T cell responses and significant inhibition of tumor growth.^304^ Notably, a computer-based de novo design caged protein nanoparticle called I53-50 has recently shown exciting results as a powerful and versatile platform for the presentation of multivalent antigens. The SARS-CoV-2 vaccine based on I53-50 showed 60 SARS-CoV-2 spike receptor binding domains in the highly immunogenic motif, which produced a neutralizing antibody titer 10 times higher than that of the control vaccine.^305^ Similarly, a nanoparticle vaccine based on I53-50 that can display 20 respiratory syncytial viruses DS-Cav1 trimer antigens induced an approximately 10-fold higher neutralizing antibody response than DS-Cav1 trimer antigens alone.^87^

#### Inorganic nanomaterials

Gold nanoparticles are one of the commonly used inorganic nanomaterials delivery systems.^306,307^ Since the physicochemical properties such as composition, size, morphology, hydrophobicity, surface charge, homogeneity, and distribution of gold nanoparticles can be precisely tuned by material synthesis and surface chemical modification to shape specific immune types, gold nanoparticles have been studied as a promising antigen delivery system in recent years.^308^ Gold nanoparticles promote antigen presentation and enhance adaptive immune responses by (1) protecting antigens from degradation and (2) forming nanovaccines of reasonable particle size to facilitate direct antigen transport to lymph nodes. In addition, Zhu et al. reported that gold nanoparticles could enhance antigen-specific antibody production by activating NLRP3 inflammasome and promoting the production of Th2-type cytokines.^309^ When gold nanoparticles were used as an adjuvant for the SARS-CoV-2 protein vaccine, they were also found to promote antigen-specific IgG production, but were unable to induce a cellular immune response sufficient to combat viral infection.^310^ Therefore, to expand the use of gold nanoparticles, they are often used in combination with additional immunostimulants or surface modified, to enhance their T-cell immune responses. Xu et al. prepared gold nanoparticles modified with PEI and found that the nanoparticles significantly promoted T-cell proliferation by activating APCs.^311^ Wang et al. coupled recombinant influenza hemagglutinin to gold nanoparticles and then coupled it to TLR5 agonist flagellin as a particle adjuvant system. Intranasal vaccination of mice with this vaccine was found to increase antigen-specific IgA and IgG levels, and to increase secretion of the cytokine IFN-γ and activation of CD8^+^ T cells.^312^ Clinically, dengue and SARS-CoV-2 vaccines using gold nanoparticles as adjuvants are being tested (NCT04935801, NCT05113862).

Similar to gold nanoparticles, mesoporous silica nanoparticles (MSNs) are also characterized by strong loading capacity and easy surface modification.^313^ Modified MSNs have been investigated as effective vaccine delivery systems. By assembling MSNs with iron oxide nanoparticles, Lee et al. formed hollow MSNs with extra-large mesopores. The surface modification was then performed using PEI, so that the gaps within the modified MSNs allowed efficient loading of various model proteins of different sizes, and could enhance antigen presentation and cross-presentation. When used as a cancer vaccine delivery system, it increased the production of antigen-specific CTLs, inhibited tumor growth in mice, and improved the survival rate of tumor-bearing mice.^314^ Li et al. reported an example of mesoporous silica/calcium phosphate composite nanoparticles loaded with tuberculin-purified protein derivative of tuberculin as adjuvants for tumor-associated antigens. The results showed that the composite particles were able to significantly inhibit the growth of tumors.^315^ MSNs-based materials have also been tested in studies as delivery systems for other antigens.^316^ It has been noted that MSNs have slowly started clinical trials as carriers for other drugs.^317^ Since MSNs have shown effectiveness as a vaccine delivery system in preclinical studies, we believe that vaccines based on MSNs as a delivery system will also be licensed for clinical trials in the near future.

With the development of materials science, material-based delivery systems have developed rapidly. It is now possible to enhance the adaptive immune responses of vaccination by protecting vaccine components and targeting them to APCs or specific lymphoid tissues through delivery systems. However, compared with immunostimulants, relatively few delivery systems have entered clinical testing. This may be due in part to the unresolved safety issues of the delivery systems. Therefore, the specific mechanism of the interaction between the delivery systems and the host immune cells or organs still needs further research to clarify its effectiveness and side effects, so as to better balance and control the relationship between the two.

## Conclusion and perspectives

In recent years, the emergence and spread of the novel coronavirus have brought great challenges to global public health in the prevention and control of diseases, which once again brings to light the importance of vaccine development. A topic that cannot be ignored in vaccine development is adjuvants, due to their ability to greatly enhance the adaptive immune responses of vaccines. At the beginning of the review, we summarize and describe the action mechanisms of adjuvants. According to the action mechanisms, adjuvants can be classified as immunostimulants and delivery systems. Immunostimulants activate APCs by targeting specific PRRs, leading to enhanced antigen-presenting and co-stimulatory signaling, which results in adaptive immune enhancement. The delivery systems target antigens to APCs or lymph nodes by prolonging the bioavailability of antigens to enhance the uptake and presentation of antigens by APCs, resulting in an enhanced adaptive immune response. Subsequently, this review elaborates on classical adjuvant platforms as well as adjuvant platforms under investigation, in the hope of informing the selection of rational adjuvants when developing a specific vaccine. In order to promote the better development of the adjuvant field, we believe that the following points may require attention.

First of all, selecting the right adjuvant to assist the antigen and improve the immune responses is an important issue in the development of new vaccines. However, this issue is complex and challenging. This is due to the fact that the response of the immune system to a given vaccine and adjuvant is highly dependent on the specific situation, and no one adjuvant is appropriate for the antigens in all cases. In conjunction with the description of adjuvants in this review, we propose here that the following factors need to be considered when selecting adjuvants for new vaccine development. For example, the routes of administration (e.g., intramuscular, mucosal, intraperitoneal),^318,319^ the type of immune responses required (antibody-biased or CD8^+^ T cell-biased, or both),^320,321^ the type of pathogens,^322^ the type of antigens (subunit antigens or mRNA antigens),^323–325^ and the stage of the diseases.^326^ In addition, the biological characteristics of the vaccine recipients may also be important, including species, ethnicity, age, medical history and genetic composition, etc.^327–332^ Moreover, the safety and economy of adjuvants also need to be taken into account. All of these factors may affect the effectiveness of vaccine adjuvants.

Moreover, considering that the combination of immunostimulants (mainly PRRs agonists) and delivery systems (especially nanoparticle delivery systems) are currently used for adjuvants development to achieve higher immune activation.^84,333^ Here, some issues need to be carefully considered. First, through our knowledge of the yellow fever virus vaccines, we know that simultaneous activation of multiple innate receptors is more effective than activation of a single receptor.^334^ Therefore, there is a great need to clarify the signaling interactions between different PRRs in order to better screen for a more effective combination of PRRs.^335^ In addition, given the differences of physicochemical characteristics between antigens and PRR agonists, and between different PRR agonists, delivery systems need to be more rationally designed to be flexible and compatible enough to deliver multiple vaccine components simultaneously.

Finally, we would also like to mention a point about classical adjuvants. The classical adjuvant has shown good biosafety in historical use. Besides, the mature research and development technologies and well-established manufacturing conditions and equipments provide a relatively easy platform for the development of new vaccines to be translated. Nevertheless, the disadvantage of classical adjuvants is their limited immune stimulation ability. In view of this, there is an urgent need for new adjuvants to make up for the shortcomings of classical adjuvants. However, the cost and time of preclinical development and clinical trials limit the conversion of new adjuvants. Therefore, we believe that while promoting the development and translation of new adjuvants, some performance optimization and formulation improvement of classical adjuvants, such as surface modification, granulation, and combination with other adjuvants that improve their immune efficacy, may be a research project with clinical value.^336,337^

In this review, we systematically summarize the mechanisms of action of adjuvants, and introduce and discuss the characteristics and application scenarios of different types of adjuvants according to their mechanisms. We expect this review to provide a reference value for further research on the mechanisms of adjuvants, rational use of existing adjuvants, and design and development of new adjuvants.
